# Prognostic value of c-Met overexpression in pancreatic adenocarcinoma: a meta-analysis

**DOI:** 10.18632/oncotarget.20392

**Published:** 2017-08-22

**Authors:** Jung Han Kim, Hyeong Su Kim, Bum Jun Kim, Jin Lee, Hyun Joo Jang

**Affiliations:** ^1^ Division of Hemato-Oncology, Department of Internal Medicine, Kangnam Sacred-Heart Hospital, Hallym University Medical Center, Hallym University College of Medicine, Seoul 07441, Republic of Korea; ^2^ Department of Internal Medicine, National Army Capital Hospital, The Armed Forces Medical Command, Sungnam 13574, Republic of Korea; ^3^ Division of Gastroenterology, Department of Internal Medicine, Dongtan Sacred-Heart Hospital, Hallym University Medical Center, Hallym University College of Medicine, Hwasung 18450, Republic of Korea

**Keywords:** c-Met, pancreatic cancer, prognostic value, meta-analysis

## Abstract

The overexpression of c-Met protein has been detected in pancreatic adenocarcinoma (PAC). However, its prognostic impact remains unclear. We performed this meta-analysis to evaluate the prognostic value of c-Met overexpression in PAC. A systematic computerized search of the electronic databases such as PubMed, Embase, and Google Scholar was carried out. From 5 studies, 423 patients who underwent surgical resection for PAC were included in the meta-analysis. Compared with patients with PAC showing low c-Met expression, patients with c-Met-high tumor had significantly worse disease-free survival (hazard ratio = 1.94 [95% confidence interval, 1.46–2.56], *P* = 0.00001) and overall survival (hazard ratio = 1.86 [95% confidence interval, 1.19–2.91], *P* = 0.006). In conclusion, this meta-analysis demonstrates that c-Met overexpression is a significant prognostic marker for poor survival in patients who underwent surgical resection for PAC.

## INTRODUCTION

Despite the recent advances in diagnostic and therapeutic modalities, pancreatic adenocarcninoma (PAC) is still among lethal malignancies with 5-year survival rates of less than 10% [1, 2]. Surgical resection with or without adjuvant therapy is the potential curative therapy for patients with a localized disease, but patients usually present with unresectable advanced diseases at the time of diagnosis. Moreover, most patients who underwent complete resection develop recurrent diseases during the course of their disease [3, 4]. For advanced or metastatic PAC, systemic chemotherapy can prolong survival compared with best supportive care, but unfortunately median overall survival (OS) was less than ten months [5, 6]. Thus, the development of more effective treatment is mandated.

With more understanding of molecular mechanisms of carcinogenesis, novel molecular agents targeting epidermal growth factor receptor, vascular epithelial growth factor receptor, or c-Met has been proposed for the treatment of PAC [7, 8]. However, the identification of biomarkers associated with response is essential to improve therapeutic outcomes of these molecular agents. Therefore, it is still necessary to accumulate our knowledge at the genomic and molecular levels.

*MET* is a proto-oncogene that encodes tyrosine kinase receptor for hepatocyte growth factor (HGF) [9]. HGF, also known as a scatter factor, binds to c-Met protein (the product of *MET* gene) and initiates auto-phosphorylation of an intracellular kinase on the beta-subunit of the receptor. This interaction allows the binding and activation of multiple signaling molecules such as Src, PI3K, Gab1, SOS, or MEK1/2 [9, 10]. This muti-faceted activation results in cellular alterations that contribute to carcinogenesis. The HGF-c-Met signaling pathway ultimately leads to tumor differentiation and proliferation, cellular invasion, angiogenesis and metastasis [11, 12]. The enhanced expression of c-Met protein has been observed in various tumors such as breast cancer [13], lung cancer [14], gastric cancer [15], colorectal cancer [16], cervix cancer [17], or hepatocellular carcinoma [18]. Several meta-analyses demonstrated that c-Met was a strong prognostic indicator of poor survival [13–17].

The overexpression of c-Met protein has also been detected in PAC [19–25]. However, most studies had a small number of patients, and its prognostic role remains unclear. We performed this meta-analysis to evaluate the prognostic value of c-Met overexpression in PAC.

## RESULTS

### Results of search

**Figure 1:**
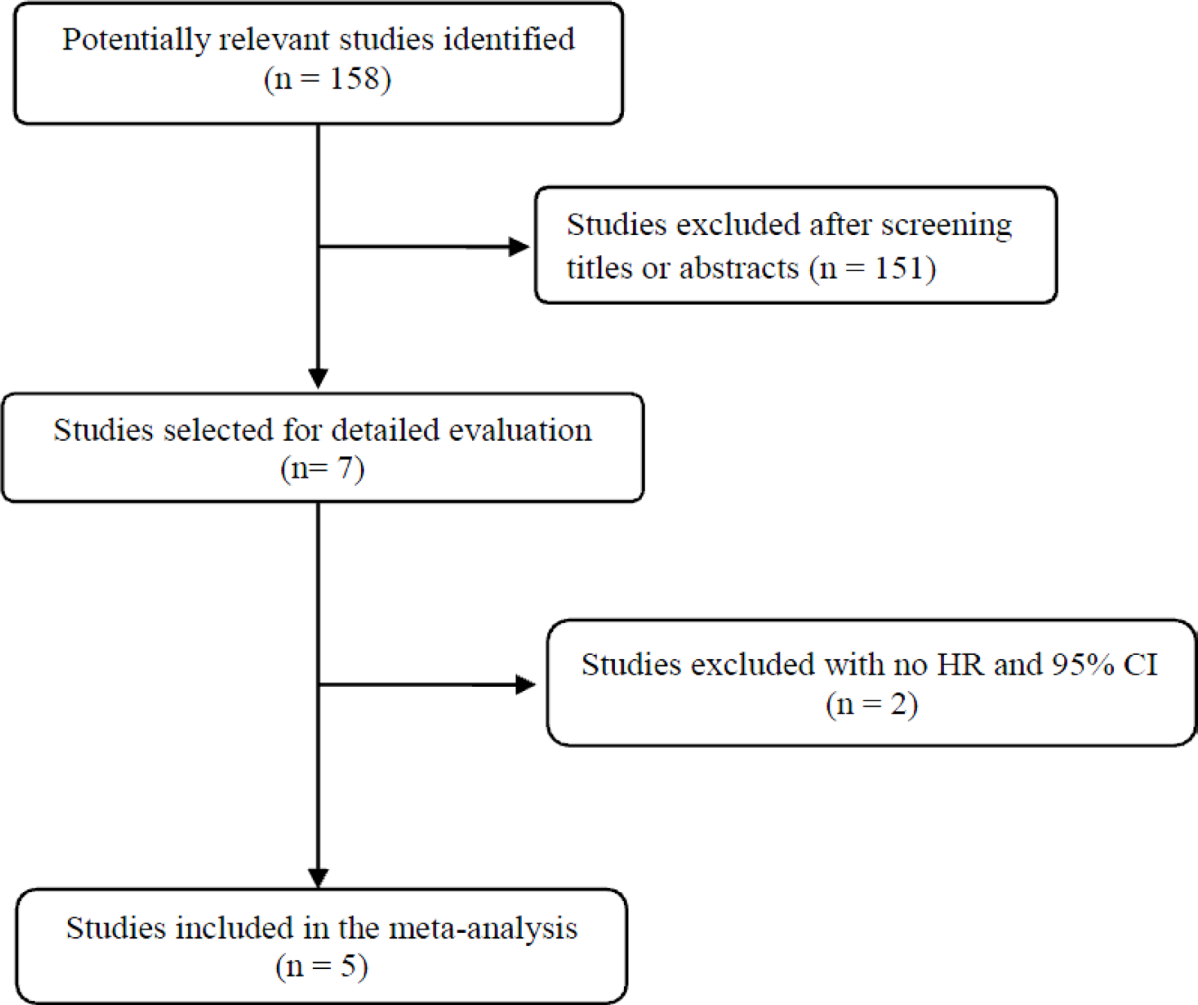
Flow diagram of search process

Figure 1 shows the flowchart of our study. A total of 158 potentially relevant studies were initially found, but 151 of them were excluded after screening the titles and abstracts. Of the remaining 7 potentially eligible studies, 2 were further excluded by the inclusion criteria because the required hazard ratio (HR) with 95% confidence interval (CI) stratified by c-Met expression were not extractable from the presented data [19, 20]. Finally, 5 studies were included in the meta-analysis [21–25].

### Characteristics of the included studies

Table 1 summarizes the main characteristics and clinical outcomes of the five included studies. All the studies were performed retrospectively in patients with PAC who underwent radical resection. From the 5 studies, 423 patients were included in the meta-analysis. In one study with 92 patients [25], 56 (60.8%) received preoperative chemoradiotherapy. Except for two studies [21, 22], three provided the data of adjuvant treatment. Out of 311 patients from the 3 studies [23–25], 214 (68.8%) received adjuvant chemotherapy with or without radiation.

**Table 1:** Summary of the five included studies

				c-Met results					
Author (year) Location	Antibody, dilution	No. of patients	IHC criteria	c-Metlow	c-Methigh	mDFS (mo) Low v high	HR for DFS (95% CI)	mOS (mo) Low v high	HR for OS (95% CI)
Ide et al., (2007) Japan	Anti-Met, clone B-2, 1:100	41	Negative: cytoplasmic staining < 30% of tumor cells. Positive (c-Methigh): ≥ 30%	24 (58.5%)	17 (41.5%)	NA	2.08 (0.72–6.05) *P* = 0.047	NA	NA
Zhu et al., (2011) China	Anti-Met, ab51067, 1:100	71	P-score: % of positive tumor cells: ≤ 10% = 1; 11–50% = 2; 51–70% = 3; ≥ 71% = 4. I-score: 0 = none; 1 = weak; 2 = moderate; 3 = strong. (c-Methigh: P-score + I-score = 4–7)	28 (39.4%)	43 (60.6%)	NA	NA	NA	2.43 (1.24–4.75) *P* = 0.010
Park et al., (2012) Korea	Anti-phospho-c-Met	88	I-score: 0 = none; 1 = weak; 2 = moderate; 3 = strong. (c-Methigh: ≥ 2 in ≥ 20% of positive tumor cells)	48 (55%)	40 (45%)	17.4 v 8.5	1.30 (0.78–2.18) *P* = 0.241	23.5 v 21.6	1.11 (0.75–1.65) *P* = 0.599
Neuzillet et al., (2015) France	Anti-Met, SP44	131	Simplified c-Met score. (c-Methigh: ≥ 20% of tumor cells with strong membrane staining)	95 (72.5%)	36 (27.5%)	20 v 9.3	2.165 (1.40–3.34) *P* = 0.0005	35 v 18.2	1.83 (1.16–2.90) *P* = 0.0098
Tomihara et al., (2017) Japan	Anti-human c-Met, 1:400	92	Semiquantititave scoring method (P-score x I-score) [26]. (c-Methigh: ≥ 7 points)	43 (46.7%)	49 (53.3%)	NA	2.58 (1.47–4.63) *P* = 0.001	NA	2.95 (1.61– 5.65) *P* = 0.0004

IHC, immunohistochemistry; P-score, proportion score; I-score, intensity score; mDFS, median disease-free survival; mOS, median overall survival; HR, hazard ratio; CI, confidence interval; NA, not available.

### c-Met expression assignation

c-Met expression was assessed by immunohistochemistry (IHC). There was a marked heterogeneity between the thresholds used to dichotomize c-Met status (c-Met^low^ or c-Met^high^). IHC criteria were briefly summarized in the Table 1. The rate of high c-Met expression ranged from 27.5% [24] to 60.6% [22].

### Impact of c-Met expression on disease-free survival

**Figure 2:**
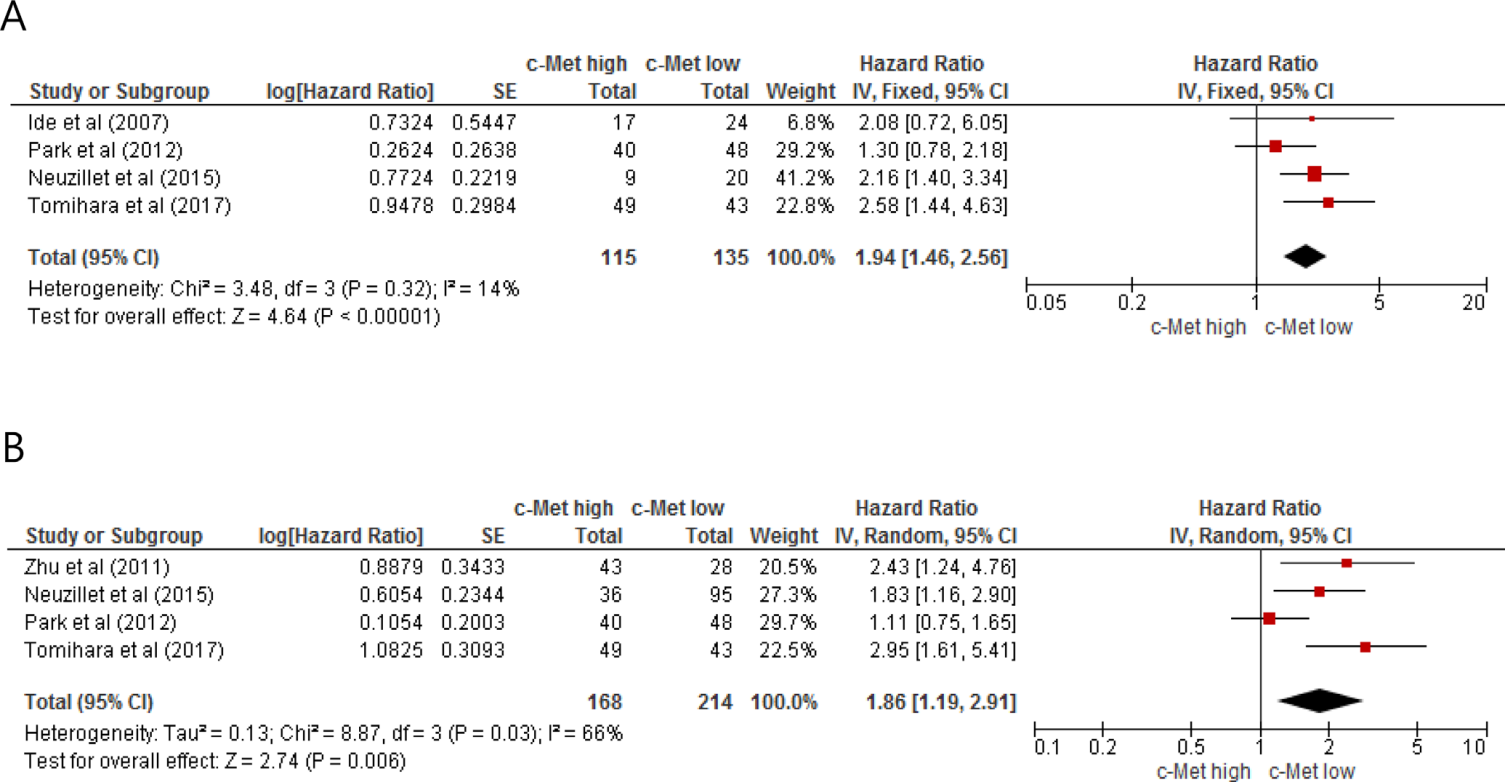
Forest plots for disease-free survival (**A**) and overall survival (**B**).

From four studies [21, 23–25], 250 patients were included in the meta-analysis of HRs for disease-free survival (DFS). Compared with patients with PAC showing low c-Met expression, patients with c-Met-high tumor showed significantly worse DFS (HR = 1.94 [95% CI, 1.46–2.56], *P* = 0.00001) (Figure 2A). The fixed-effect model was used because there was no significant heterogeneity (*X*^2^ = 3.48, *P* = 0.32, *I*^2^ = 14%).

### Impact of c-Met expression on overall survival

From four studies [22–25], 382 patients were included in the meta-analysis of HRs for OS. Patients with c-Met-high PAC showed significantly shorter OS than those with c-Met-low PAC (HR = 1.86 [95% CI, 1.19–2.91], *P* = 0.006) (Figure 2B). The random-effect model was adopted because of significant heterogeneity across the studies (*X*^2^ = 8.87, *P* = 0.03, *I^2^* = 66%).

### Publication bias

**Figure 3:**
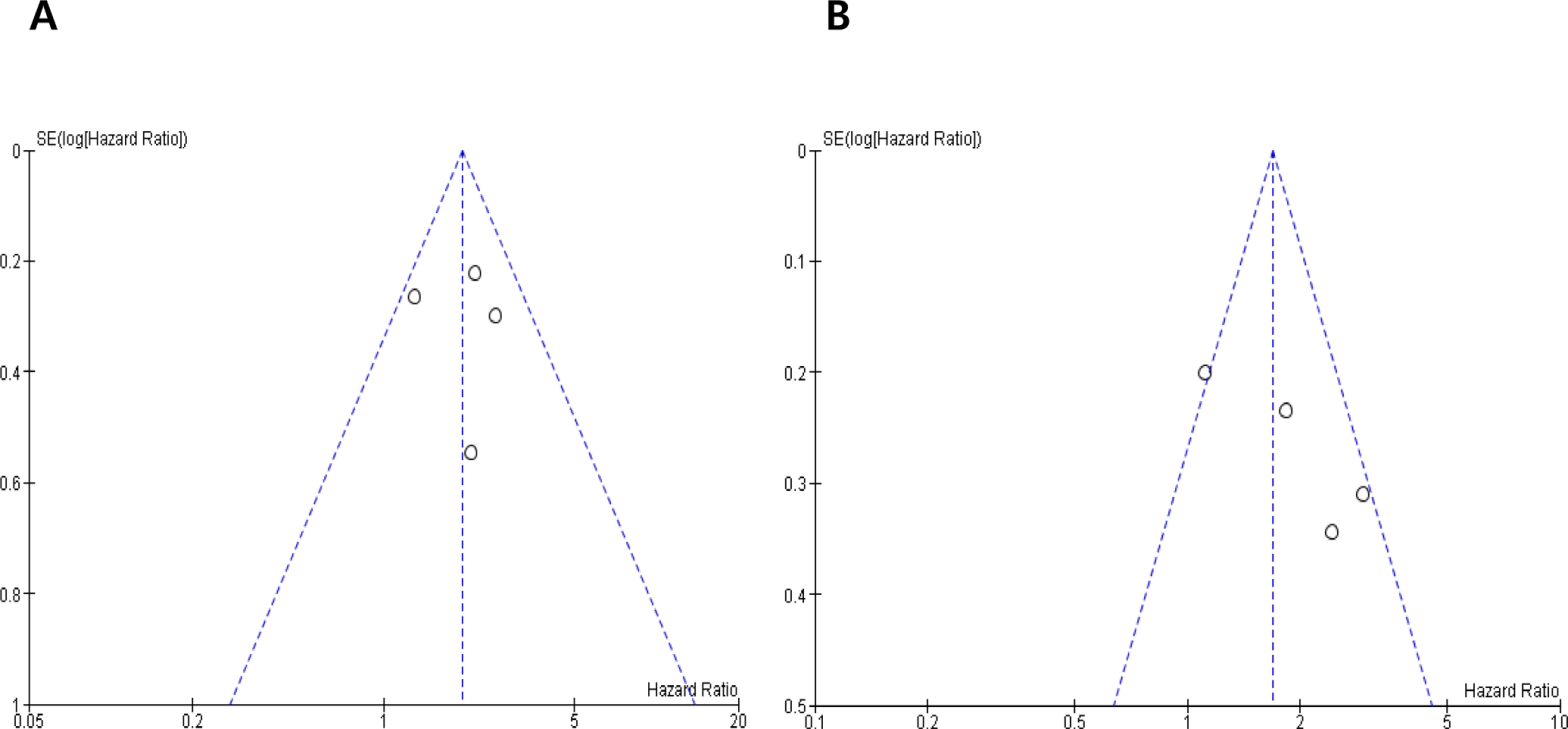
Funnel plots for publication bias regarding disease-free survival (**A**) and overall survival (**B**).

Visual inspection of the funnel plots for DFS and OS showed symmetry, indicating there were no publication biases (Figure 3A and 3B).

## DISCUSSION

In this meta-analysis, we evaluated the prognostic impact of c-Met overexpression in patients with resected PAC. The results show that high c-Met expression is associated with significantly poor DFS or OS. To our knowledge, this is the first meta-analysis suggesting that c-Met overexpression represent an adverse prognostic marker in patients with PAC.

PAC shows unfavorable prognosis with the most aggressive tumor biology. The traditional post-operative prognostic factors such as tumor size, lymph node involvement, or status of resection margin are insufficient to predict patients with a high risk of recurrence or metastasis. Therefore, the identification of reliable predictive markers and potential therapeutic targets is essential to guide individual treatment strategies and improve prognosis in patients with PAC. c-Met has been proven to play a critical role in the pathogenesis and progression of many tumor types [9–12].

The enhanced expression of c-Met has also been observed in PAC [19–26]. Because most studies had a small number of patients and adopted various IHC scoring methods, however, they could not draw a consensus regarding the prognostic value of c-Met. In an early study by Furukawa *et al*., patients with PAC showing diffuse staining for c-Met showed better OS than those with tumors showing no or focal staining (*P* = 0.026 by log-rank test). However, this study had a very small sample size (27 patients for OS comparison) and classified patients by the c-Met positivity or negativity, not by the c-Met expression status (low or high). In the current meta-analysis, we only included studies comparing survivals (DFS or OS) according to the c-Met expression status. Patients with c-Met-high PAC showed significantly shorter DFS (HR = 1.94, *P* < 0.00001) and OS (HR = 1.86, *P* = 0.006), compared with those with c-Met-low tumor. Our results indicate that high c-Met expression is a significant prognostic marker for poor survival in patients with resected PAC.

Multiple studies also demonstrated that high expression of c-Met was associated with poor survival in various cancers [13–18]. Thus, interference with c-Met activation may provide an effective therapeutic approach for cancers with c-Met overexpression [27]. Several c-Met inhibitors are currently under active investigation in various cancer types [10, 28–31]. The efficacy of c-Met-targeting agents has been associated with high c-Met expression in non-small-cell lung cancer and hepatocellular carcinoma [28, 29]. Therefore, patients with PAC overexpressing c-Met protein might be good candidates for c-Met inhibitors. Indeed, it has been demonstrated that targeting c-Met impairs tumor growth and improves activity of gemcitabine in PAC [29–32].

However, the major challenge for clinical development of c-Met inhibitors is that there are no standardized methods and criteria for c-Met overexpression. A variety of methods such as IHC, Western blot, fluorescence *in situ* hybridization, or real-time quantitative PCR are currently used for assessing c-Met status [13]. In this meta-analysis, the included studies adopted the various IHC methods with the different criteria for c-Met overexpression. The discrepancies in the prognostic value of c-Met overexpression in the previous reports with PAC might be attributable to the different c-Met scoring methods. Therefore, the definition of a reliable guideline for c-Met status is an essential prerequisite for assessing the prognostic role of c-Met expression and developing c-Met inhibitors in solid tumors.

Our study has inherent limitations that should be noted. First, the meta-analysis included a small number of studies with a limited sample size. Second, the included studies were all retrospectively performed. Third, of the five studies, four were conducted in Asia. Finally, as we already mentioned, IHC criteria to stratify c-Met status were various among studies.

In conclusion, our meta-analysis demonstrates that c-Met overexpression is a significant prognostic marker for poor survival in patients who underwent surgical resection for PAC. However, larger studies using standardized methods are still needed to verify the prognostic role of c-Met expression in PAC.

## MATERIALS AND METHODS

### Publication searching strategy

This study was conducted according to the Preferred Reporting Items for Systematic Reviews and Meta-Analyses (PRISMA) guidelines [33]. We performed a systematic computerized search of the electronic database PubMed, Embase, and Google Scholar (up to April 2017). The search was carried out using the following keywords: ‘c-Met’ or ‘Met’ and ‘pancreatic cancer’ or ‘pancreas neoplasm’ or ‘pancreatic adenocarcinoma’. The related articles function in the PubMed was also used to identify all relevant articles.

### Inclusion criteria

Eligible studies should meet the following inclusion criteria: (i) patients had a diagnosis of PAC; (ii) DFS and/or OS were analyzed by c-Met expression status; (iii) HRs with 95% CIs for DFS or OS were reported or could be calculated from the data provided; (iv) papers were written in English.

### Data extraction

Data extraction was carried out independently by two investigators (BJK and HSK). If these two authors did not agree, other investigators (JHK and HJJ) were consulted to resolve the dispute.

The following data were extracted from all eligible studies: first author's name, year of publication, country, number of patients, tumor stage, treatment, methodology of IHC, the criteria used to dichotomize c-Met expression as ‘high’ or ‘low’, and HR with 95% CIs for DFS or OS.

### Statistical analysis

Statistical values used in this meta-analysis were obtained directly from the original articles. When papers had no HR and 95% CI, the Engauge Digitizer version 9.1 was used to estimate the needed data from Kaplan-Meier curves. The effect size of DFS and OS was combined through HR and its 95% CI. Heterogeneity among studies was estimated using the chi-square-based Cochran's Q statistic and *I*^2^ inconsistency test: *P* < 0.1 and *I*^2^ > 50% indicated the presence of significant heterogeneity. The fixed-effects model (Mantel-Haenszel method) was selected to calculate the pooled HR when substantial heterogeneity was not observed. When significant heterogeneity was detected across studies, we adopted the random-effects model (DerSimonian-Laird method).

The RevMan version 5.2 was used to combine the data. The plots show a summary estimate of the results from all the studies combined. The size of the squares represents the estimate from each study and reflects the statistical ‘weight’ of the study (relative contribution to the summary estimate). Results are presented as forest plots with diamonds representing estimate of the pooled effect and the width of diamond representing its precision. The line of no effect is number one for binary outcomes, which depicts statistical significance if not crossed by the diamond [34]. All reported *P*-values were two-sided and *P* < 0.05 was considered statistically significant. Publication bias was assessed graphically by the funnel plot method [35].
